# Clinical study of “Jiaotai Pill” combined with head massage with 5-tone rhythm on insomnia patients of heart-kidney disharmony type

**DOI:** 10.1097/MD.0000000000032645

**Published:** 2023-01-06

**Authors:** Ruiqian Guan, Limin Pan, Zhiguo Yu, Zhao Liu, Qingchun Shi, Jingjing Li

**Affiliations:** a Heilongjiang University of Traditional Chinese Medicine, Harbin, China; b The Second Affiliated Hospital of Heilongjiang University of Traditional Chinese Medicine, Harbin, China; c First Affiliated Hospital of Heilongjiang University of Traditional Chinese Medicine, Harbin, China.

**Keywords:** 5 elements music, head massage, insomnia, Jiaotai pill, massage

## Abstract

To explore the curative effect of “Jiaotai Pill” combined with head rhythmic massage consistent with 5-tone rhythm on insomnia of heart-kidney disharmony type.

**Methods:**

Sixty patients with insomnia in massage clinic and ward were randomly divided into treatment group A (30 cases) and treatment group B (30 cases). Patients in group A were treated with traditional head massage combined with oral estazolam tablets. Group B was treated with “Jiaotai Pill” combined with head rhythmic massage therapy consistent with 5-tone rhythm. After 2 weeks of treatment, the scores of Hamilton Anxiety Scale, Pittsburgh Sleep Quality Index, Insomnia Severity Index and Traditional Chinese Medicine Symptom Scale, as well as the expression changes of interleukin (IL)-6 and IL-8 in serum were compared between the 2 groups before and after treatment.

**Results:**

After 2 weeks of treatment, the total effective rate of group B was 93. 33%, which was significantly higher than that of group A (66. 67%) (*P* < .05). After treatment, the scores of Hamilton Anxiety Scale, PQSI, insomnia severity index and traditional Chinese medicine symptom scores were significantly decreased in both groups, and the decrease in group B was more significant than that in group A (*P* < .05). After treatment, the serum levels of IL-6 and IL-8 were significantly decreased in both groups, and the decrease in group B was greater than that in group A, the difference was statistically significant (*P* < .05).

**Conclusion:**

The overall efficacy of Jiaotai Pill combined with head massage therapy consistent with 5-tone rhythm is significantly better than that of traditional massage combined with 5-element music therapy for insomnia patients with heart-kidney disharmony.

## 1. Introduction

Insomnia is called insomnia in traditional Chinese medicine (TCM), and its main pathogenesis in TCM is imbalance of yin and yang. The earliest extant documents that can be verified are the silk books before the Tang Dynasty, *Moxibustion on the Eleven Meridians of Yin and Yang* and *Moxibustion on the Eleven Meridians of Foot and Arm*.^[1]^ It is also recorded in the *Canon of Internal Medicine* that head massage has a long history of treating insomnia, which can be traced back to the earliest record in *Miraculous Pivot: Meridians* that “the Yangming meridians of the hands and feet are distributed on the forehead and face, and the Shaoyang meridians of the hands and feet are distributed on the side of the head,” and the Conception Vessel and Governor Vessel are the qi of yin and yang of the whole body and run in the middle of the head, so it is feasible to treat insomnia by head massage. The prescription of “Jiaotai Pill” first appeared in the *Treatise on Spleen and Stomach* written by Li Dongyuan in the Jin and Yuan dynasties. In *On the Injury Caused by Excessive Drinking*, but it is not a medicine for the disharmony between the heart and the kidney. Wang Shixiong’s *Brief Prescription of Four Subjects*, Anshen, in the Qing Dynasty, clearly proposed that *Coptis chinensis* and *Cinnamomum cassia* should be used together to treat disharmony between the heart and the kidney, which was named Jiaotai pill. The “Jiaotai Pill” used in this paper is based on the main prescription in Wang Shixiong’s “Brief Prescriptions for Four Subjects: Tranquilizing the Mind.”^[2]^

The head rhythmical 5-element music massage therapy is a rhythmical music therapy technique which is based on the traditional head massage technique, based on the “five-tone” treatment theory of TCM, and refers to the modern music therapy concept. In clinical practice, the research team has been recognized and praised by the majority of patients, and has achieved good therapeutic effect, but there has been a lack of objective basis and indicators. The clinical observation of this treatment plan and the traditional treatment is as follows.

## 2. Objects and methods

### 2.1. Object

All the cases were from the outpatient and inpatient wards of The Second Affiliated Hospital of Heilongjiang University of Traditional Chinese Medicine. In this study, according to the diagnostic criteria of the disease, inclusion criteria, and exclusion criteria of the subjects. With the equation: N1 = N2 = (μα + μβ) 22p (1−P)/ (p1−p2) 2, p = (P1 + P2)/2. The sample size was estimated, α = 0.05, β = 0.1, μ0.05 = 1.64, μ0.1 = 1.28 from the value table, P1 = 90%, P2 = 57% from the literature, and n = 30 from the formula. Therefore, at least 30 subjects were needed in each group, and at least 60 patients with insomnia of heart-kidney disharmony type were recruited to complete the clinical trial. From June 2021 to May 2022, 60 insomnia patients aged 40 to 60 years who were admitted to our hospital and agreed to be enrolled were randomly divided into treatment group A and treatment group B, of which 1 case was dropped out in each group, and 1 case was terminated in treatment group A, which had no statistical significance. Finally, 30 cases were enrolled in each group. Treatment group A (14 males and 16 females) had an average age of (47.2 ± 4.2) years and a course of disease of 3.89 ± 1.22 weeks, while treatment group B (15 males and 15 females) had an average age of (47.7 ± 5.2) years and a course of disease of 3.79 ± 1.12 weeks, which were basically the same, without statistical significance and comparable (see Table 1). Before treatment, there was no statistical significance in the general information score of the 2 groups of patients, that is, *P* > .05. Both groups of patients passed the ethical review and agreed to be enrolled.

**Table 1:** Baseline condition of 2 groups of patients.

Group	Gender (F/M)	Age (yr)	Course of disease (wk)
Treatment group A	16/14	47.2 ± 4.2	3.89 ± 1.22
Treatment group B	15/15	47.7 ± 5.2	3.79 ± 1.12

### 2.2. Diagnostic criteria

#### 2.2..1. Diagnostic criteria of Western medicine.

According to Diagnostic and Statistical Manual of Mental Disorders^[3]^ formulated by American Psychiatric Association, it is difficult to fall asleep for a long time (duration ≥30 minutes), light sleep, poor sleep quality, easy to wake up (wake up >2 times at night), early to wake up, difficult to fall asleep again after waking up, mental fatigue during the day, and total sleep time <6 hours. Duration ≥30 days.

#### 2.2..2. TCM diagnostic criteria.

According to the National Administration of TCM, the diagnostic criteria of insomnia in the diagnostic and therapeutic criteria of TCM^[4]^: light person enters sleep difficulty or sleep and wake up easily, wake up hind insomnious, successive 3 weeks above, heavy person sleepless all night; it is often accompanied by headache, dizziness, palpitation, restlessness and dreaminess; this disease often has a history of improper diet, emotional disorders, excessive work and rest, excessive thinking, and weakness after illness; and through various systems and laboratory examinations, no other organic pathological changes that hinder sleep were found. TCM syndrome differentiation standard: refer to the formulation of insomnia of central kidney disharmony type in Internal Medicine of TCM.^[4]^ Main symptoms: vexation, insomnia, difficulty in falling asleep, palpitation and dreaminess; secondary symptoms: dizziness and tinnitus, soreness and weakness of the waist and knees, hectic fever and night sweat, feverish sensation in the chest, palms and soles, red tongue with little coating, and thready and rapid pulse.

### 2.3. Inclusion criteria

Those who met the above diagnostic and dialectical criteria of insomnia; total score >7 according to PSQI; all patients should stop the relevant therapeutic drugs 1 week before inclusion; and patients should be included in the study voluntarily and sign the relevant ethical informed consent.

### 2.4. Exclusion criteria

Those who did not meet the diagnostic criteria or inclusion criteria; temporary insomnia caused by environmental and external factors; insomnia caused by various organic diseases; insomnia caused by sleep disorders; and patients with mental illness diagnosed by specialists.

### 2.5. Termination criteria

Serious adverse reactions or special physiological changes or other unexpected events occur during the trial, and the subject is not suitable to continue the trial; the patient’s condition deteriorates during the trial, and emergency measures must be taken because of possible danger; the patient is unwilling to continue the trial during the trial; and the subject violates the clinical trial protocol during the treatment.

### 2.6. Treatment

Source of the main study cases: the source of the cases were 60 patients with insomnia in the massage clinic and ward of our hospital who met the criteria for incorporation. The patients were randomly divided into 2 groups, 30 cases in group A and 30 cases in group B. The specific research methods are as follows:

#### 2.6..1. Treatment group A.

On the basis of conventional treatment in the massage department, the patients were given traditional head massage treatment. The massage technique was the commonly used acupoint pressing method on the head. Baihui, Sishencong and other acupoints were pressed respectively for about 1 minute each point, and then the sweeping method, kneading method, pressing and kneading method were used. The frequency of the manipulation was 60 to 100 times per minute, and the strength of the manipulation was based on the patient’s tolerance. At the same time, listen to the music of the 5 elements, and the volume should be comfortable and relaxed. According to the different TCM syndromes of insomnia patients, different modes of music were selected, including Gong, Shang, Jiao, Hui and Yu modes. The type of disharmony between the heart and the kidney chooses the music of quotient-sign mode.^[5]^ Patients and physicians also listened to music with headphones and observed and recorded the treatment effect. Each patient was treated for 15 minutes each time, twice a day, 5 days a week, 2 weeks as a course of treatment, and oral estazolam tablets (GYZZ H37023047) 1 tablet (1mg/tablet) once a day, half an hour after dinner, for 1 course of treatment.

#### 2.6..2. Treatment group B.

On the basis of the routine treatment in the massage department, the patients were given the head rhythmic massage treatment consistent with the 5-tone rhythm. The strength of the manipulation was based on the patient’s tolerance, and the frequency of the manipulation was the same as that of the music. That is to say, the patient’s massage is accompanied by 5-element music therapy, in which the frequency and rhythm of the therapist’s manipulation are completely consistent with the style and rhythm of the selected music, and the therapist and the patient listen to the same group of music at the same time. With the headphone shunt, the 4 lines simultaneously play the therapeutic tracks as to meet the need of consistency of the doctor’s technique and the rhythm. The volume should be comfortable and relaxed for the patient. According to the different TCM syndromes of insomnia patients, different modes of music were selected, including Gong, Shang, Jiao, Hui and Yu modes. The type of disharmony between the heart and the kidney chooses the music of quotient-sign mode.^[5]^ The therapeutic effects were observed and recorded. Each patient was treated for 15 minutes, twice a day, 5 days a week, 2 weeks a course of treatment, and oral administration of Jiaotai Pill 1 pill, half an hour after dinner, a total of continuous treatment for 1 course of treatment.

### 2.7. Observation index

#### 2.7.1.

The clinical efficacy was formulated according to the 2012 edition of the Criteria for Diagnosis and Efficacy of TCM Diseases and the Pittsburgh Sleep Quality Index (PSQI) score, which was divided into 4 grades: recovery, markedly effective, effective and ineffective. Cure: symptoms disappeared, PSQI score ≤7; markedly effective: symptoms relieved, PSQI score ≤7; effective: symptoms relieved, PSQI scores >7; ineffective: symptoms did not improve, PSQI scores >7. Total effective rate = (30−number of ineffective cases)/30 × 100%.

#### 2.7.2.

PSQI, proposed by Dr Buysse et al, a psychiatrist at the University of Pittsburgh in the US in 1989, is a questionnaire used to assess the subjective sleep quality of subjects in the last month. It consists of 18 entries. Subjective sleep quality, sleep latency, sleep duration, sleep efficiency, sleep disorder, hypnotic use, and daytime dysfunction. The total score >7 was taken as the criterion of poor sleep quality. The scores of each component and the cumulative total score of PSQI were recorded before and after treatment. The total score ranged from 0 to 21. The higher the score, the worse the sleep quality. When the total score was ≤7, the sleep quality was better.

#### 2.7.3.

Insomnia Severity Index (ISI) scale: the scale is divided into 2 parts: the severity of insomnia and the degree of satisfaction with sleep. The higher the score, the more serious the insomnia, which was evaluated by patients themselves before and after treatment.^[6]^

#### 2.7.4.

Hamilton Anxiety Scale (HAMA) evaluates the severity of anxiety, which is divided into 2 parts: physical anxiety and mental anxiety. The higher the score, the more serious the anxiety. The patients were self-evaluated before and after treatment.^[6]^

#### 2.7.5.

TCM syndrome integral scale: assessed according to the Technical Guidelines for Clinical Research of New TCM Drugs with Syndromes.^[7]^ Main symptoms (vexation and insomnia, difficulty in falling asleep, palpitation and dreaminess) and secondary symptoms (dizziness and tinnitus, soreness and weakness of waist and knees, hectic fever and night sweat, vexation with feverish sensation in chest, palms and soles) were graded into 0, 2, 4, 6 and 0, 1, 2, 3 according to absence, mild, moderate and severe, respectively. The higher the score, the more serious the clinical sign and symptom was.

#### 2.7.6.

Observe the expression of interleukin (IL)-6 and IL-8 in the serum of patients in the 2 groups, unit: PG/mL; the reference interval of IL-6 (0–10) and IL-8 (0–60) were determined by immunofluorescence method, using the kit manufactured by Wondfo.

### 2.8. Statistical methods

SPSS 22.0 software package was used for statistical analysis of the data in this study. The measurement data in line with normal distribution was represented by X2, and if it did not meet the normal distribution, it was described by the median; if it met the normal distribution, it was tested by *t* test, and if it did not meet the normal distribution then it was tested by rank sum test. If *P* < .05, the difference was statistically significant.

## 3. Results

### 3.1. Comparison of clinical efficacy between the 2 groups

After 2 weeks of treatment, the total effective rate of group B was 93.33%, which was significantly higher than that of group A (66.67%), and the difference was statistically significant (*P* < .05), as shown in Table 2.

**Table 2:** Comparison of therapeutic effects of 2 groups of patients with insomnia of heart-kidney disharmony type.

Grouping	Number of cases	Heal	Remarkable effect	Valid	Not valid	Total effective rate
Treatment group A	30	4	6	10	10	66.67%
Treatment group B	30	19	6	3	2	93.33%

### 3.2. Comparison of PSQI scores between the 2 groups

Before treatment, there was no significant difference in PSQI scores between the 2 groups (*P* > .05); after treatment, the PSQI scores and total scores of the 2 groups were significantly decreased (*P* < .05), and the decrease in group B was higher than that in group A, and the difference was statistically significant (*P* < .05), see Table 3.

**Table 3:** Comparison of PSQI scores between the 2 groups of patients with insomnia of heart-kidney disharmony type.

Group	Treatment group A (n = 30)		Treatment group B (n = 30)	
Before treatment	After treatment	Before treatment	After treatment
Sleep quality	2.48 ± 0.70	2.01 ± 0.69a	2.43 ± 0.65	1.24 ± 0.76ab
Time to fall asleep	2.29 ± 0.98	1.90 ± 0.84a	2.30 ± 0.76	1.21 ± 0.54ab
Sleep time	2.45 ± 0.80	2.12 ± 0.73a	2.37 ± 0.74	1.53 ± 0.60ab
Sleep efficiency	2.42 ± 0.97	2.13 ± 0.75a	2.19 ± 1.02	1.25 ± 0.88ab
Sleep disorders	1.69 ± 0.80	1.03 ± 0.77a	1.74 ± 0.71	0.61 ± 0.32ab
Daytime function	2.47 ± 0.81	1.97 ± 0.99a	2.51 ± 0.77	1.30 ± 0.75ab
Total PSQI score	13.78 ± 2.50	10.11 ± 3.36a	13.54 ± 2.37	6.13 ± 2.27ab

Compared with the same group before treatment, aP < .05; compared with the treatment group A, bP < .05.

PSQI = Pittsburgh Sleep Quality Index.

### 3.3. Comparison of the scores of ISI and HAMA between the 2 groups

Before treatment, there was no significant difference in the scores of ISI and HAMA between the 2 groups (*P* > .05); after treatment, the scores of ISI and HAMA in the 2 groups were significantly decreased (*P* < .05), and the decrease in group B was more significant than that in group A (*P* < .05), see Table 4.

**Table 4:** Comparison of ISI and HAMA scores between the 2 groups of patients with insomnia of heart-kidney disharmony type.

Group	ISI		HAMA	
Before treatment	After treatment	Before treatment	After treatment
Treatment A n = 30	18.86 ± 5.48	13.24 ± 4.75a	16.92 ± 8.60	11.74 ± 7.32a
Treatment group B n = 30	19.05 ± 5.58	8.17 ± 4.25ab	17.15 ± 8.73	8.36 ± 6.55ab

Note: Compared with the same group before treatment, aP < 0.05; compared with the treatment group A, bP < .05.

HAMA = Hamilton Anxiety Scale, ISI = Insomnia Severity Index.

### 3.4. Comparison of TCM symptom scores between the 2 groups

The TCM symptom scores of the 2 groups after treatment were significantly lower than those before treatment (*P* < .05), and the reduction degree of the treatment group B was better than that of the treatment group A, and the difference was statistically significant (*P* < .05), see Table 5.

**Table 5:** Comparison of TCM symptom scores between the 2 groups of insomnia patients of heart-kidney disharmony type.

Project	Treatment group A		Treatment group B	
Before treatment	After treatment	Before treatment	After treatment
Upset and sleepless	3.47 ± 1.63	1.93 ± 1.34a	3.53 ± 1.46	1.27 ± 1.11ab
Difficulty falling asleep	2.40 ± 1.52	1.67 ± 1.18a	2.47 ± 1.63	0.93 ± 1.01ab
Palpitation and dreaminess	2.40 ± 1.61	1.47 ± 1.28a	2.53 ± 1.38	0.87 ± 1.01ab
Dizziness and tinnitus	2.60 ± 1.50	1.40 ± 1.19a	2.67 ± 1.60	0.80 ± 1.00ab
Soreness and weakness of the waist and knees	1.77 ± 0.73	1.13 ± 0.68a	1.73 ± 0.69	0.77 ± 0.63ab
Hot flashes, night sweats	2.10 ± 0.80	1.27 ± 0.74a	2.13 ± 0.73	0.77 ± 0.73ab
Red tongue with little fur	0.80 ± 0.41	0.50 ± 0.51a	0.77 ± 0.43	0.23 ± 0.43ab
Thready and rapid pulse	0.87 ± 0.35	0.63 ± 0.49a	0.83 ± 0.38	0.33 ± 0.48ab
Total score	1.640 ± 3.55	1.000 ± 3.19a	16.67 ± 3.31	5.97 ± 2.70ab

Note: Compared with the same group before treatment, aP < .05; compared with the treatment group A, bP < .05.

TCM = traditional Chinese medicine.

### 3.5. Comparison of serum detection indexes of 2 groups of patients

Before treatment, there was no significant difference in serum IL-6 and IL-8 levels between the 2 groups (*P* > .05); after treatment, the levels of IL-6 and IL-8 in the 2 groups were significantly lower than those before treatment (*P* < .01), and the levels of IL-6, IL-8 in the treatment group B were significantly lower than those in the treatment group A (*P* < .05), see Table 6.

**Table 6:** Comparison of serum IL-6 and IL-8 in 2 groups of patients with insomnia of heart-kidney disharmony type.

Group	Before treatment		After treatment	
Serum inflammatory factor	IL-6	IL-8	IL-6	IL-8
Treatment group A	16.53 ± 2.35	72.03 ± 4.34	7.82 ± 1.14a	46.83 ± 6.58a
Treatment group B	16.47 ± 3.43	71.77 ± 4.52	3.26 ± 0.82ab	34.37 ± 7.48ab
*T* value	0.097	−0.227	17.786	−6.851
*P* value	.923	.821	<.001	<.001

Note: Compared with the same group before treatment, aP < .01; compared with the treatment group A, bP < .05.

IL = interleukin.

## 4. Discussion

Insomnia can be chronic (>3 months) or short-term (<3 months).^[8]^ The previously used terms “primary” and “secondary” insomnia and different subsets of chronic insomnia, such as “psychophysiological insomnia,” have been replaced by the broader term “insomnia,” but are still sometimes encountered in the medical literature. Insomnia is common in people with mental or physical health conditions that affect sleep, but it can still be diagnosed as a disease. Insomnia can cause problems such as inability to concentrate and low work efficiency. With the development of the disease, it can even lead to memory decline, metabolic obstruction, irritability and other conditions,^[9]^ seriously affecting the health and quality of life of patients. The neurobiological mechanisms of insomnia are complex. From the proposal of hyperarousal mechanism to the release of neurotransmitters in different brain regions, from neuroendocrine to neuroimmune mechanism, from cellular level to gene and molecular level, many factors are closely linked and jointly participate in the regulation of sleep-wake cycle.^[10]^ Estazolam is one of the widely used hypnotics in clinic. Although it can prolong sleep time, it cannot change the quality of sleep, but also produce dizziness, lethargy, memory loss and other adverse reactions.^[11]^ Insomnia is often a chronic process. However, medication for sleep problems is generally recommended for short-term use.^[12,13]^ Due to the limitations of pharmacotherapy, including potential dependence and tolerance under long-term use,^[14,15]^ and the recognition of excessive anxiety factors in the insomnia trajectory, non-pharmacological approaches have received increasing attention in the past decade. In particular, cognitive behavioral therapy for insomnia (CBT-I) has become the most prominent non-pharmacological treatment and is recommended as a first-line treatment for insomnia. The American Academy of Sleep Medicine^[16,17]^ and the American Medical Association^[18]^ recommend CBT-I as a first-line treatment for chronic insomnia in adults. CBT-I is a multi-component treatment that targets behavioral, cognitive, and physiological factors that contribute to insomnia and aims to modify and change maladaptive behaviors and distorted beliefs about sleep and insomnia.^[19,20]^ It typically involves 4 to 8 weekly sessions (50–90 minutes) led by a trained therapist covering the following topics: stimulus control, sleep restriction, relaxation techniques, cognitive therapy, and sleep hygiene education. Non-drug therapies such as acupuncture, massage and music therapy are typical manifestations of CRT-I.

Compared with sedative and hypnotic drugs, acupuncture at Shu points, massage and other TCM therapies have achieved obvious advantages in clinical diagnosis and treatment because of their high safety, no dependence, less toxic and side effects, and remarkable effect. For a long time, the combination of 5-tone therapy and massage has been based on listening to 5-element music while massaging.^[21]^ And the head massage therapy which is adopted by us and is consistent with the 5-tone rhythm is a rhythmical treatment technique which is based on the head massage technique in the traditional massage technique, combined with the 5-tone treatment theory of the TCM and is integrated and innovated by taking the modern music treatment theory as a train of thought and is consistent with the rhythm of the 5 element music. The main characteristic of this technique is that the change of rhythm is consistent with the rhythm of music, which is essentially different from the fixed massage rhythm in traditional techniques. The earliest theory and mechanism of music therapy in China is the form of combining 5 tones with 5 elements, which appeared in the period of Neijing. Zhu Zhenheng of Yuan dynasty clearly pointed out that “music is also medicine.”^[5]^ The physiological characteristics of the 5 Zang organs correspond to the 5 tones. It is recorded in the *Four Diagnostic Methods* that “the spleen corresponds to the palace, and its sound diffuses to slow the lung, its sound promotes to clear the liver, its sound calls to long the heart, its sound is strong to clear the kidney, and its sound is deep and thin.” It is precisely because the characteristics of the 5 tones correspond to the physiological functions of the 5 Zang organs that there is a corresponding relationship between the 5 internal organs and the 5 tones.^[22]^ Zhang Jiebin, a physician in the Ming Dynasty, wrote in his book *Lei Jing Fu Yi · Lv yuan*, “Music is the harmony of heaven and earth.” Rhythm is the righteousness of heaven and earth, and the sound of human beings,” and it is believed that music “can connect heaven and earth with the gods”.^[23]^ The expression of music and regulating yin and yang can be traced back to Neijing, which regards the balance of yin and yang as the basis of physical and mental health. In Taiping Jing of the Eastern Han Dynasty, the theory of yin and yang is used to explain the significance of health preservation and the origin of music. The book holds that the law of the dynamic and static correspondence of all things in the universe and the mutual generation of yin and yang is the foundation of music development.^[24]^ The explanation of music with the principle of yin and yang can be summarized as follows: high is yang, low is yin; major is yang, minor is yin; strong is yang, weak is yin; strong is yang and soft is yin, and the sound of golden leather is yang and the sound of silk and wood is yin.^[25]^ Many studies have shown that the good use of music can affect and treat people’s emotions.

The accurate record of Jiaotai Pill comes from Wang Shixiong In Brief Prescriptions of Four Subjects, Tranquilizing the Mind, the full text is “Sheng Chuan Lian Wu Qian, Cinnamon Heart Wu Fen, grinding, white honeyed pills, hollow light salt soup, treatment of heart and kidney disharmony, anxiety and insomnia, named Jiaotai Pill.” It not only explains the drug composition and dosage of Jiaotai Pill, but also the method of taking the drug and the main symptoms of the disease, which is an important exclusive Chinese medicine prescription for insomnia of heart-kidney disharmony type. At the same time, some studies have shown that Jiaotai Pill can act on the circadian rhythm control center and sleep-wake center of the body through related pathways.^[26]^ Modern pharmacological studies have found that the rat model of water extract of Jiaotai Pill was induced by intragastric administration of chlorophenylalanine, and the concentrations of tumor necrosis factor-α, IL-6, IL-1β and other cytokines in serum and brain tissue of insomnia rats were detected, and the contents of sleep-related neurotransmitters were detected. The results showed that compared with the control group, the contents of tumor necrosis factor- α, IL-6, IL-1β, 5-hydroxytryptamine, glutamicacid and ACH in the brain and serum of insomnia rats in the Jiaotai Pill group changed significantly.^[27–29]^ “Jiaotai Pill” is often used to treat depression and insomnia in clinic. In recent years, animal experimental studies have shown its antidepressant effect. Clinical researchers have carried out clinical studies on depression and insomnia of disharmony between heart and kidney in elderly patients with cerebral infarction and achieved good results.^[30]^ The attempt of this clinical study belongs to the blank of this professional field, which provides a valuable and feasible scheme for further research.

To sum up, “Jiaotai Pill” combined with head massage therapy consistent with 5-tone rhythm has exact clinical efficacy, which can significantly improve the PSQI, ISI, HAMA and TCM symptom scores of insomnia patients, effectively reduce the expression of serum IL-6 and IL-8 factors, and then improve the discomfort symptoms of patients. The clinical effective rate was 93.33%, which was significantly different from 66.67% in group B (*P* < .05). “Jiaotai Pill” combined with the head massage which is consistent with the 5-tone rhythm can give full play to the advantages of traditional Chinese massage and 5-element music in the treatment of insomnia, which is a good, painless and acceptable manipulation treatment, and is worthy of clinical promotion.

## Author contributions

**Conceptualization:** Ruiqian Guan.

**Data curation:** Ruiqian Guan, Limin Pan, Zhiguo Yu, Zhao Liu, Qingchun Shi, Jingjing Li.

**Formal analysis:** Ruiqian Guan.

**Funding acquisition:** Ruiqian Guan.

**Investigation:** Ruiqian Guan.

**Methodology:** Ruiqian Guan.

**Project administration:** Ruiqian Guan.

**Resources:** Ruiqian Guan.

**Software:** Ruiqian Guan.

**Supervision:** Ruiqian Guan.

**Validation:** Ruiqian Guan.

**Visualization:** Ruiqian Guan.

**Writing – original draft:** Ruiqian Guan.

**Writing – review & editing:** Ruiqian Guan.
